# Breastfeeding at Any Cost? Adverse Effects of Breastfeeding Pain on Mother–Infant Behavior

**DOI:** 10.3390/biology12050636

**Published:** 2023-04-22

**Authors:** Maayan Abargil, Merav Irani, Nathalie klein Selle, Shir Atzil

**Affiliations:** 1 The Department of Psychology, The Hebrew University of Jerusalem, Jerusalem 9190501, Israel; 2 Criminology Department, Bar-Ilan University, Ramat Gan 5290002, Israel

**Keywords:** breastfeeding, bonding, maternal behavior, infant behavior, affect, regulation, ARCS, allostasis

## Abstract

**Simple Summary:**

Breastfeeding is encouraged worldwide due to its nutritional and bonding benefits, but more attention needs to be given to the potential psychological challenges it poses to new mothers. This study investigated whether breastfeeding pain relates to mothers’ and infants’ bonding behavior. Seventy-one mothers with varying levels of breastfeeding pain were videotaped with their infants during face-to-face interactions. We found that mothers with severe breastfeeding pain express less affect and less infant-directed gaze during interactive moments of engagement and play than mothers with no or moderate pain. Infants of mothers experiencing pain also express less affect and more mother-directed gaze than those of mothers not experiencing pain. These findings suggest that maternal pain can interfere with the behaviors of both mothers and infants, potentially impacting development and bonding. Since the mother–infant dyad is a codependent allostatic unit, the allostatic challenges of one partner can influence both partners. Therefore, nutritional advances should be considered along with additional allostatic consequences of breastfeeding to ensure the well-being of both mothers and infants.

**Abstract:**

Breast milk is considered the ideal infant nutrition, and medical organizations encourage breastfeeding worldwide. Moreover, breastfeeding is often perceived as a natural and spontaneous socio-biological process and one of the fundamental roles of new mothers. While breastfeeding is beneficial, little scientific consideration has been given to its potential psychological challenges. Here, we investigate the phenomenon of breastfeeding pain in mothers and its association with maternal and infant behavioral regulation. During the postpartum weeks, the mother–infant dyad can be considered one allostatic unit directed at infant regulation and development. We hypothesize that pain comprises an allostatic challenge for mothers and will thus impair the capacity for dyadic regulation. To test this, we recruited 71 mothers with varying levels of breastfeeding pain and videotaped them with their infants (2–35 weeks old) during spontaneous face-to-face interactions. We quantified the individual differences in dyadic regulation by behaviorally coding the second-by-second affective expressions for each mother and infant throughout their interactions. We tested the extent to which breastfeeding pain alters affect regulation during mother–infant interactions. We discovered that mothers with severe breastfeeding pain express less affective expressions and less infant-directed gaze during interactive moments of engagement and play than mothers with no or moderate pain. Moreover, infants of mothers experiencing pain during breastfeeding express less affective expressions and more mother-directed gaze while interacting with their mothers than infants of mothers who are not in pain. This demonstrates that the allostatic challenge of maternal pain interferes with the behavioral regulation of both mothers and infants. Since the mother–infant dyad is a codependent allostatic unit, the allostatic challenges of one partner can impact the dyad and thus potentially impact child development, bonding, and mother and infant well-being. The challenges of breastfeeding should be considered in addition to the nutritional advances.

## 1. Introduction

Breast milk is widely considered the ideal nutrition for infants, advancing the health of both infants and mothers and promoting infant development [1]. For example, breast milk proteins decrease the risk of obesity, adiposity [2], and diabetes [3]. More extended periods of breastfeeding reduce the prevalence of Crohn’s disease [4], augment the infant’s immune system [5], and reduces cases of allergies and asthma [6]. Moreover, breastfeeding affects infants’ neural function [7] and anatomy. Infants exclusively breastfed for at least three months showed increased brain white matter in frontal cortical regions [8,9]. Breastfeeding is also associated with improved social and cognitive development [10], intelligence [11], memory [12], and language in infants [13]. Breastfeeding is advantageous for mothers too, and is associated with a reduced risk of breast cancer and diabetes [14,15]. Furthermore, some studies associate breastfeeding with improved maternal mood [16], sleep [17], maternal care [18], and mother–infant bonding [19,20,21]. Consequently, all major medical organizations recommend breastfeeding as a significant source of infant nutrition for at least one year [22,23,24,25].

Currently, most studies on breastfeeding have focused on the benefits for the infant and mother [26,27]. Yet, despite such consensual recommendations, women often choose not to breastfeed throughout their first postpartum year. While most women in the United States and Europe initiate breastfeeding, many mothers wean the infant within the first few postpartum weeks [27]. In 2019–2020, 83.9% of American women reported starting breastfeeding, yet only 25.8% continued breastfeeding exclusively after six months [28]. Shorter durations of breastfeeding were associated with psychological hardship, lower self-efficacy, and lower self-confidence [29]. In addition, a negative experience of breastfeeding is associated with symptoms of depression [30], a sense of struggle and loneliness [27], altered maternal mood, and sleep disturbances [31].

One of the main reasons to stop breastfeeding is pain [32]. Breastfeeding pain affects up to 68% of breastfeeding mothers [33,34] and is perceived as highly distressing [35]. Much research addressed breastfeeding pain, mainly concerning ankyloglossia (or tongue-tie) [36,37]. This research tested the effects of pain on breastfeeding quality [31,38] and breastfeeding outcomes [39,40,41]. Motivated to improve the feeding outcome and the maternal experience [34,42,43], several studies addressed the treatments for breastfeeding pain involving medication [44,45] or frenotomy [43,46]. While this research is mainly directed toward the outcome of feeding, no study we know addresses the effects of breastfeeding pain on the mother–infant relationship.

Pain can modulate and be modulated by social interactions. Positive social interactions alleviate pain [47,48,49,50,51,52], whereas negative social relations are associated with increased pain [49,50,51,53,54,55]. The opposite direction is also evident: pain can impact social behavior toward an individual in pain by eliciting prosocial behaviors in both humans and rodents [47,48,53,56]. Pain can also change the social tendencies of the individual in pain. At a physiological level, chronic pain promotes an allostatic load [57,58] characterized by neuroendocrine dysregulation, fatigue, and impaired mental and physical performance, which can adversely impact social interactions [59,60,61,62]. Specifically, pain was demonstrated to alter the expressions and regulation of affect [50,63,64]. Given the importance of expression and regulation of affect during social interactions, pain can interfere with the ability to interact, which is particularly relevant in the mother–infant bond.

At the beginning of life, the mother–infant relations focus on the infant’s survival and regulation. The newborn infant depends entirely on the mother (or the primary caregiver) to regulate most fundamental physiological processes [65,66,67,68,69,70]. This process is called *Allostasis* and refers to the ongoing adjustment of an individual’s internal milieu necessary for survival, growth, and reproduction [71,72,73,74]. To regulate the infant’s allostasis, mothers must be attuned to their infant’s behavioral cues and constantly address them to correct even subtle allostatic disturbances [75]. Accordingly, adjusted maternal care is essential for optimal child well-being and development [76,77,78].

Affect expressions and gaze are key behavioral features exchanged during social interactions [79,80]. Mothers attune their affective expressions to moments of mutual gaze [81,82] when infants are engaged in the interaction [83]. Consequently, with development, infants increase the duration of visual fixation on the mother’s face and express more affect during an interaction [84]. Infants’ positive and negative affective expressions indicate their regulation [85]. Accordingly, mothers tend to be sensitive and responsive to the infants’ affective expressions [85,86,87], resulting in the mutual facial coupling of affective expressions [84,88]. This positive feedback loop of gaze and facial expressions is essential for the infants’ affective development [75], teaching infants to synchronize and regulate their affect during social interactions. In this sense, mutual gaze and affective facial expressions are essential for mothers to infer the infant’s allostatic needs, provide attuned allostatic care, and promote infants’ learning and development [89,90].

Regulating infants through attuned interactions is demanding for mothers and requires heightened availability and resources [91]. Mothers must be attentive and constantly adjust their behavior to notice and address every subtle allostatic cue the infant communicates [86]. Such a demanding process is susceptible to regulatory challenges posed to the mother herself, such as pain, illness, and psychopathology [92,93]. Pain is a regulatory challenge that depletes physiological and psychological resources [94,95]. Moreover, the consistent presence of pain or the expectancy of pain reduces engagement in different activities and increases a sense of helplessness [94,96,97]. Breastfeeding takes hours throughout the day, especially in the first months of life [98]. If painful, breastfeeding can be a source of ongoing pain and pain expectancy. Mothers who experience pain in every feeding struggle while breastfeeding, and continuously dread the next feeding, increasing the allostatic load.

Here, we test whether breastfeeding pain adversely affects mothers’ and infants’ behavior during free interaction. We hypothesize that since pain is an allostatic challenge for mothers, breastfeeding pain will adversely impact the mothers’ behavioral regulation of infants. Specifically, we predict that mothers who suffer from breastfeeding pain will show altered affective expressions during interactions and be less responsive to infant cues. Moreover, since infant affective communication is contingent on maternal affective communication [99,100,101,102,103,104], we further predict that infants of mothers with breastfeeding pain will demonstrate altered affective communication during interactions.

## 2. Methods

### 2.1. Participants

A total of 71 mother–infant dyads participated in the study: 50 dyads where the mothers were experiencing varying levels of breastfeeding pain (from no pain to severe pain) and a control group of 21 non-breastfeeding mothers. Mothers with breastfeeding pain were recruited through a lactation consultant, while other mothers were recruited from an early childhood center or through an ad post on Facebook. Mothers’ ages ranged from 22 to 39 years. Infants’ ages ranged from 2 to 35 weeks (breastfeeding pain: $\bar{age}$ = 12.02 weeks, σ = 8.88; no pain: $\bar{age}$ = 13.40 weeks, σ = 8.97; not breastfeeding: $\bar{age}$ = 17.96 weeks, σ = 9.47). In our cohort, the maternal income level ranged from lower than average (10% of mothers) to somewhat lower than average (19% of mothers) to average (17% of mothers) to slightly higher than average (37% of mothers) to higher than average (17% of mothers). The maternal relationship statuses were in a relationship (12% of mothers), married (83% of mothers), or single (5% of mothers). Infants were born via vaginal delivery (82% of mothers), cesarean section (14% of mothers), or vacuum extraction delivery (4% of mothers). The mean number of children in the household was 1.56 children (sd = 0.92), with a mean of 3.11 rooms (sd = 0.71). The mean years of education were 17.18 years (sd = 1.39). The target sample size was assessed with power analysis (G*Power software [105]). Since no research had addressed this question, a moderate effect size was estimated based on previous studies that reported a moderate to high correlation between maternal emotional pain and maternal bonding behavior (r values ranging between 0.31 and 0.873) [106,107,108]. Therefore, given a power of 0.95 and estimated moderate effect size in a linear regression of 0.35 for the correlation between breastfeeding pain and maternal behavior, the target sample size was 50 dyads with breastfeeding mothers. After collecting the data, we excluded one mother from the “breastfeeding without pain” group since she reported having prior breastfeeding pain. Therefore, the results include only 19 breastfeeding mothers with no breastfeeding pain. The Institution Review Board approved the study, and mothers signed an informed consent form before participating. Families were remunerated for their participation.

### 2.2. Procedures

Mothers experiencing breastfeeding pain were recruited through lactation consultants, who asked mothers experiencing pain to participate in the research. Mothers without pain or mothers not breastfeeding were recruited through social media. Participation included questionnaires and a video recording of a two-minute free interaction with the infant. Videos were captured in the families’ homes or their local community center. Trained research personnel filmed the interaction based on our previous research [83,85,109,110]. Mothers were instructed to freely interact with their infants as usual, without any restrictions, specific toys, or guidelines; the videos did not include breastfeeding. This setting represents natural mother–infant interactions [111,112], allowing each mother to determine her setup, interaction choices, and tendencies without constraints. The free mother–infant interactions were then imported to the lab and behaviorally coded and analyzed by trained personnel using the Affect Regulation Coding Scheme (ARCS) [113].

### 2.3. Behavior

#### 2.3.1. ARCS—Affect Regulation Coding Scheme

Using the ARCS, trained coders coded the mothers’ and infants’ second-by-second affective expressions during the interactions.

The ARCS is a coding scheme that enables us to trace the *valence*, *expression intensity*, and *gaze* separately for mothers and infants for each second of the interaction. *Valence* represents the moment-by-moment valence of facial expression: positive, neutral, or negative. *Expression intensity*  represents the changing effort of the facial expression, ranging from no facial effort to strong effort. *Gaze and attention* trace the mothers’ and infants’ gaze and attention, ranging from an unfocused gaze to a directed gaze to switching glances (Table 1).

#### 2.3.2. Inter-Rater Reliability

The inter-rater reliability was conducted for each variable separately based on 10–12% of the interactions [85,114,115,116,117]. Krippendorff’s alpha test was used to estimate the inter-rater reliability between coders, given its accuracy in assessing the level of agreement between raters for categorical variables of multiple levels [118]. The inter-rater reliability score for maternal behavior was Krippendorff’s α = 0.835 and for the infant behavior, Krippendorff’s α = 0.855, which is considered moderate to high [118] (see Supplementary Anaysis S1, Table S1 for reliability scores of each variable).

After the initial coding, we used an in-house toolbox using MATLAB R2016a (The MathWorks, Natick, MA, USA) and Python (version 3.7) to compute the mean and dynamic changes in the three variables of valence, expression intensity, and gaze during the interaction. The scores were calculated as such:

1. General Valence: averaged 120 s of the interaction (N1).
  $$\bar{Valence}=\frac{\sum {Valence}_{i}}{N1}$$
2. General Expression Intensity: averaged 120 s of the interaction (N1).
  $$\bar{Expression\;intensity}=\frac{\sum {Expression\;intensity}_{i}}{N1}$$
3. The average duration in focused gaze: the average time mothers maintained focused gaze. N2 = the number of focused gaze events across the interaction.
  $$\bar{focused\;gaze}=\frac{\sum {Time\;in\;focused\;gaze}_{i}}{N2}$$

For more information on the ARCS and its implementation, email the corresponding author.

### 2.4. Breastfeeding Pain

#### 2.4.1. Breastfeeding Questionnaire: Mothers Completed a Questionnaire about Their Current Breastfeeding Pain Experience. Scores Started from 0 (No Pain at All) and Gradually Increased to 10 (Extremely Intense and Unbearable Pain)

We divided all breastfeeding mothers into three groups, a priory defined by the questionnaire score:

1. No pain: N = 19, questionnaire score of 0. These mothers reported that they had not experienced any breastfeeding pain since the second postnatal week.
2. Moderate pain: N = 16, questionnaire score of 1–4.
3. Severe pain: N = 14, questionnaire score of 5–10.
4. Additionally, we recruited 21 mothers who were not breastfeeding as a control group.

#### 2.4.2. Computing the Association between Breastfeeding Pain and Maternal Behaviors

We tested the correlation between the degree of breastfeeding pain and three maternal behaviors:

1. Duration of directed gaze;
2. Expressed valence;
3. Affective expression intensity.

We applied two analyses:

(1) A Pearson correlation between the degree of breastfeeding pain and the three maternal behavior variables while controlling for infants’ age. Given the three dependent variables, Bonferroni correction for multiple hypotheses was applied.
(2) A one-way ACNOVA to test the group differences in the three maternal behaviors across the four groups (no pain = 19, moderate pain = 16, severe pain = 14, not breastfeeding = 21) while controlling for infants’ age. We further calculated group differences using post hoc LSD.

#### 2.4.3. Maternal Behavior during Infant Engagement and Distress across Pain Groups

The ARCS provides second-by-second measures of dyadic affect, which enables us to assess in a dynamic way how maternal behavior unfolds during specific events of infant affective regulation. Maternal behavior was evaluated during two types of infant regulatory events: engagement in play and distress.

Infant Engagement: Events of infant engagement were defined as infants transitioning from neutral to positive valence. We determined all events where infants transitioned from neutral to positive valence across all the free interaction videos and aligned them in time (153 events).

Infant Distress: Events of infant distress were defined as infants transitioning from neutral to negative valence. We located all events where infants transitioned from neutral to negative valence across all the free interaction videos and aligned them in time (123 events).

Maternal valence, intensity, and gaze were averaged across all participants for a given second during events of engagement and distress (separately) and compared between pain groups with repeated-measure ANCOVA. A repeated-measure general linear model (ANCOVA) was conducted to test the temporal changes in maternal valence and expression intensity across the four breastfeeding groups while controlling for infant age differences (five groups X seven time points). We further calculated specific differences using the post hoc LSD.

#### 2.4.4. Computing the Association between Breastfeeding Pain and Infant Behaviors

We tested the correlation between the degree of breastfeeding pain and three infant behaviors:

1. Duration of directed gaze;
2. Expressed valence;
3. Affective expression intensity.

We applied two types of analyses:

(1) A Pearson correlation between the degree of breastfeeding pain and the three maternal behavior variables while controlling for infants’ age.
(2) A one-way ANCOVA to test the group differences in the three maternal behaviors across the four breastfeeding groups (no pain = 19, moderate pain = 16, severe pain = 14, not breastfeeding = 21) while controlling for infants’ age. We further calculated group differences using the post hoc LSD.

## 3. Results

### 3.1. The Association between Maternal Breastfeeding Pain and Maternal Behaviors

**Figure 1:**
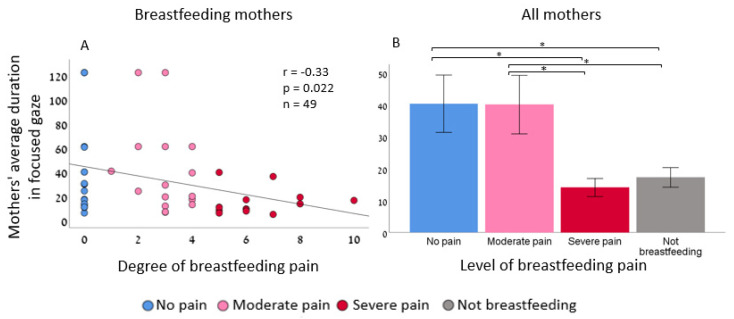
Mothers with severe breastfeeding pain maintain a less focused gaze during face-to-face interaction with the infant. (**A**) Partial Pearson’s correlation between the degree of breastfeeding pain and mothers’ average time spent in focused gaze revealed a significant correlation after controlling for infants’ age differences. (**B**) Maternal-focused gaze was significantly different across the four breastfeeding groups. Post hoc LSD showed that mothers with severe pain maintained a less focused gaze compared to mothers with no breastfeeding pain (Cohen’s d = −0.94, *p* = 0.010, 95% CI [−46.65, −6.73]) and mothers with moderate pain (Cohen’s d = −0.92, *p* = 0.015, 95% CI [−46.74, −5.29]). Interestingly, mothers who do not breastfeed maintain a less focused gaze compared to mothers with no pain (Cohen’s d = −0.73, *p* = 0.024, 95% CI [−39.44, −2.81]) and mothers with moderate pain (Cohen’s d = −0.70, *p* = 0.038, 95% CI [−39.77, −1.13]), * *p* < 0.05.

Mothers with severe pain maintained a less focused gaze compared to mothers with no pain or with moderate pain (Pearson r = −0.33, *p* = 0.022 two-tailed, *n* = 49, 95% CI [−0.49, −0.15]) (Figure 1A), one-way ANCOVA, F (3,65) = 3.91, *p* = 0.013, η^2^ = 0.15 (Figure 1B)). There was no significant association between the degree of breastfeeding pain and affect intensity (Pearson r = −0.14, *p* 0.337 two-tailed, *n* = 49, CI [−0.39, 0.16]) or with valence (Pearson r = −0.025, *p* = 0.868 two-tailed, *n* = 49, 95% CI [−0.25, 0.29]) (Supplementary Figure S1). Given the three dependent variables, Bonferroni correction for multiple hypotheses testing yielded a *p*-value of 0.016, and the effect remained marginally significant (*p* = 0.02). However, when including only mothers who experienced pain in the analysis, the correlation was higher (Pearson r = −0.47, *p* = 0.011 two-tailed, *n* = 30, 95% CI [−0.65, −0.25]).

### 3.2. The Effect of Breastfeeding Pain on Maternal Affective Reactivity during Infant Engagement and Distress

**Figure 2:**
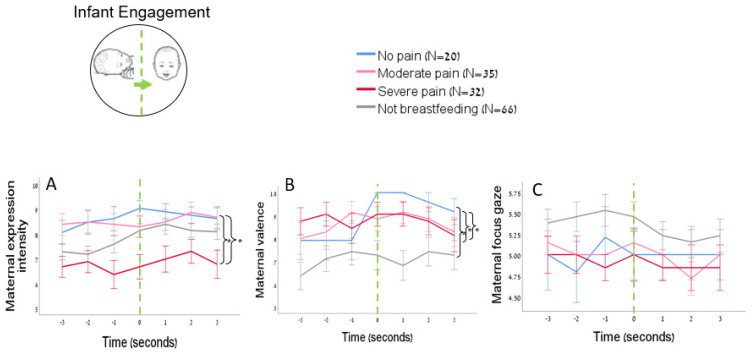
Mothers with severe breastfeeding pain demonstrated weaker affective expressions during playful interactions with the infant. The *X* axis represents time, where events of positive infant engagement in play are temporally aligned on “0” and marked by the dashed green line. (**A**) Mothers with severe breastfeeding pain (in red) were less expressive during positive interactions with their infants than mothers with no and moderate breastfeeding pain. (**B**) Mothers with no breastfeeding pain (in blue) were more responsive to the infant when they engaged in play by shifting up their positive valence just before the infants shifted up their positive valence. (**C**) Pain did not affect the maternal gaze during events of infant engagement, * *p* < 0.05.

Maternal affective expression during infant engagement: There was a significant group effect of pain on maternal affective expressions during events of infant engagement, where infants transition from neutral to positive valence (Mix ANCOVA, F(3,148) = 4.98, *p* = 0.003, η^2^ = 0.092). Post hoc LSD analyses showed that mothers who experienced severe breastfeeding pain displayed less intense facial expressions while interacting with their infant in positive engagements events compared to mothers with no breastfeeding pain (Cohen’s d = −0.91, *p* = 0.007, 95% CI [−3.43, −0.39]) and mothers with moderate breastfeeding pain (Cohen’s d = −0.80, *p* = 0.005, 95% CI [−3.01, −0.40]) (Figure 2A).

Maternal valence during infant engagement: There was a significant group effect on maternal valence (Mix ANCOVA, F(3,148) = 5.85, *p* = 0.001, η2 = 0.11). Post hoc LSD analyses showed that mothers who did not breastfeed demonstrated less positive valence compared to mothers with no breastfeeding pain (Cohen’s d = −0.77, *p* = 0.003, 95% CI [−0.35, −0.07]), mothers with moderate pain (Cohen’s d = −0.63, *p* = 0.003, 95% CI [−0.29, −0.06]), and mothers with severe pain (Cohen’s d = 0.70, *p* = 0.001, 95% CI [−0.32, −0.08]) (Figure 2B).

Maternal gaze during infant engagement: There was no significant group effect of pain on maternal gaze during events of infant engagement (Mix ANCOVA, F(3,148) = 2.57, *p* = 0.0561).

There was no significant group effect of pain on maternal behavior during events of infant distress in any of the dependent variables: expression intensity: Mix ANCOVA, F(3, 118) = 0.75, *p* = 0.525; valence: Mix ANCOVA, F (3,118) = 0.25, *p* = 858; gaze: Mix ANCOVA, F (3,118) = 267, *p* = 0.51 (Figure 2C).

### 3.3. The Association between Maternal Breastfeeding Pain and Infant Behaviors

**Figure 3:**
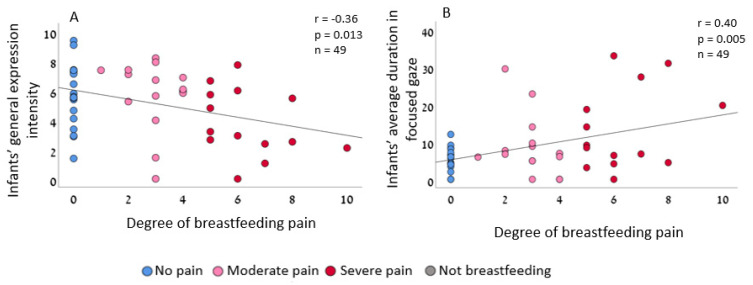
Infants of mothers with severe breastfeeding pain expressed weaker affective expressions and maintained a longer focused gaze during mother–infant interactions. (**A**) There was a significant association between the degree of maternal breastfeeding pain and infant expression intensity (Pearson r = −0.36, *p* = 0.013 two-tailed, *n* = 49, 95% CI [−0.58, −0.08]). (**B**) A significant association existed between the degree of maternal breastfeeding pain and infants’ focused gaze (Pearson r = 0.4, *p* = 0.005 two-tailed, 95% CI [0.125, 0.632]).

Next, we tested the effect of maternal breastfeeding pain on infants’ affective expression and gaze. Interestingly, infants of mothers with severe pain expressed weaker affective expressions during mother–infant interaction (Figure 3A) and maintained longer focused gaze during mother–infant interaction (Figure 3B). There was no significant association between maternal pain and infants’ expressed valence (Pearson r = 0.25, *p* = 0.089; two-tailed, *n* = 49, 95% CI [−0.035, 0.459]). Given the three dependent variables Bonferroni correction for multiple hypothesis testing is applied, *p* = 0.016, and the effect remained significant.

## 4. Discussion

The current research demonstrates that severe breastfeeding pain is associated with altered behavior of both mothers and infants during an interaction. Mothers with severe breastfeeding pain maintain a less focused gaze and communicate weaker positive affective expressions during infants’ engagement and play than mothers with no pain or moderate pain. Breastfeeding pain is also associated with infants’ behavior, as infants express weaker facial expressions and maintain a longer focused gaze compared to infants of mothers with no pain or moderate pain. This suggests that an allostatic challenge to mothers, such as pain, can interfere with the behavioral mechanism of affective communication in early childhood and is thus a potential risk factor for child development and mother–infant well-being.

Pain can lead to disengagement in social interactions [96,97]. Here, we found that in mothers, this was evident only during playful moments. There is a differential effect of pain on maternal response to the infant’s distress and play: mothers suffering from breastfeeding pain were less responsive during infant engagement and play. They expressed positive valence with significantly less intensity. However, breastfeeding pain did not affect maternal response during the infant’s distress. Infants communicate distress to signal allostatic challenges [85]. This highlights that infants’ allostatic signals priorly guide maternal behavior, and only when infants do not communicate distress do maternal allostatic challenges play a role in driving behavior. This also signifies the methodological need to separately inspect positive and negative moments of interactions, which is a central feature of the ARCS method.

Mothers with severe pain had difficulty maintaining a focused gaze during face-to-face interactions. This suggests that severe breastfeeding pain interferes with the mother’s ability to fully engage with her infant while interacting. Maternal gaze is a fundamental component of maternal-sensitive responding during a face-to-face interaction [90], which enables mothers to signal availability, establish mutual engagement, and regulate infant arousal [89,90]. Thus, this behavioral alternation can have downstream effects on infants’ behavior.

Accordingly, maternal pain affects both maternal behavior and infant behavior: infants of mothers with pain demonstrated less affective expressions during interactions. Infants are highly sensitive to adult faces, notably the caregiver gaze [119]. During face-to-face interactions, mothers typically attune to and complement the infant’s expressions with exaggerated mimicry, which in turn enhances the infant’s social communication [101]. In contrast, when parents look away during a face-to-face interaction, infants’ affective cues decline [120]. This suggests that the effect of maternal breastfeeding pain on reduced infant affective expressions could result from an altered maternal responsivity during an engagement. Future research is needed to test this on a larger sample.

While mothers in pain maintain a less focused gaze, infants of mothers with severe breastfeeding pain hold a longer focused gaze compared to infants of mothers with no or moderate pain. Gaze behavior is one of the earliest regulatory channels infants develop- through their gaze, infants control the amount and type of stimulation they perceive [91,121]. In cases of “reduced maternal feedback, such as the “still face” paradigm [122] or cases of maternal depression [123], infants often respond with an increased gaze, seeking social engagement [124]. Like depression and the “still face” experience, breastfeeding pain is associated with reduced maternal responsivity and gaze. This may elicit the infant’s increased gaze as an approach-seeking compensation mechanism to gain more information and engage the unengaged mother during interactions.

The current research has some limitations. Studying human mothers and infants does not allow for an experimental inducement of pain or other allostatic challenges or to test their causal effect on maternal and infant behavior. Future research in larger samples of both human and non-human animals that directly test the impact of allostatic interference on mothers and infants is needed to establish the role of mutual allostasis regulation in the mother–infant dyad. Moreover, this research did not include longitudinal developmental assessments of breastfeeding under severe pain on the long-term mother–infant bond and infant development.

Breastfeeding has many benefits, including promoting close mother–infant contact and social-emotional development [19,125]. Moreover, breastfeeding can enhance maternal behaviors, such as gazing at the infant and more affectionate responses [126]. Since breastfeeding is seen as essential in creating an intimate and close relationship between the mother and her infant [127], women likely feel pressured to breastfeed even under severe pain. However, here we demonstrate that breastfeeding under pain may pose adverse outcomes to both mothers and infants. Given the allostatic codependence between mothers and infants, optimal care, well-being, and child development depend on the allostasis of both mothers and infants.

## 5. Conclusions

This research demonstrates the potential negative impact of severe breastfeeding pain on the mother–infant dyad. The findings suggest that maternal pain can lead to disengagement during playful interactions, resulting in altered affective communication and reduced maternal gaze, which in turn can affect infant behavior. Optimal care, well-being, and child development depend on the allostasis of both mothers and infants; breastfeeding under severe pain may pose adverse outcomes. These findings highlight the importance of addressing maternal pain during breastfeeding to promote care and development. Future research is needed to explore the long-term impact of maternal pain and additional allostatic challenges on the mother–infant bond and infant development.

## Figures and Tables

**Table 1:** **ARCS—Affect Regulation Coding System.** Trained coders coded the second-by-second social signals of valence, the intensity of the affective expression, and the gaze during free interaction. Each variable was coded separately for each mother and infant per second.

Behavioral Coding	Coding Values
Valence	Negative/Neutral/Positive
The intensity of affective expression	No facial effort
	Mild facial effort
	Strong facial effort
Gaze	Unfocused gaze
	Directed gaze
	Glancing

## Data Availability

ARCS method and implementation can become freely available for research use by emailing the corresponding author.

## Supplementary Materials

The following supporting information can be downloaded at: https://www.mdpi.com/article/10.3390/biology12050636/s1. Table S1. The average inter-rater reliability between coders, using Krippendorff’s alpha test. Analysis S1. Confirming the consistency of each measurement across the interaction. Figure S1. The association between maternal breastfeeding pain and maternal expression intensity and valence. (A) Partial Pearson correlation between the degree of breastfeeding pain and maternal expression intensity while controlling for infants’ age. (B) Maternal expression intensity in the four pain groups, one-way ANCOVA, F (3,65) = 0.291 *p* = 0.832. (C) Partial Pearson’s correlation between the degree of breastfeeding pain and maternal valence while controlling for infants’ age. (D) Maternal valence in the four pain groups, one-way ANCOVA, F (3,65) = 0.844 *p* = 0.475.
