# Supplementary material for: Breastfeeding at Any Cost? Adverse Effects of Breastfeeding Pain on Mother–Infant Behavior
Source: Biology (Basel). 2023 Apr 22;12(5):636. doi: 10.3390/biology12050636 (PMC10215422; doi:10.3390/biology12050636)
Supplement: Supplementary file 1 [file biology-12-00636-s001.zip › biology-2320930-supplementary.pdf]

Table S1. The average inter-rater reliability between coders, using Krippendorff's alpha test

	Mathers	Infants
Valence	α =0.830	α=0.846
Expression intensity	α=0.847	α=0.820
Gaze	α=0.829	α=0.898

Analysis S1. Confirming the consistency of each measurement across the interaction

We tested the distribution of each behavioral measurement in two random bins of 60 seconds. Kolmogorov-Smirnov test confirmed the similarity of the two distributions of each measurement for each dyad, supporting the reliability of these measures.

Infant average arousal score: K-S= 0.551, p=0.922

Infant valence K-S= 0.521, p=0.949

Parent average arousal score K-S= 1.183, p=0.122

Parent valence K-S= 0.417, p=0.995

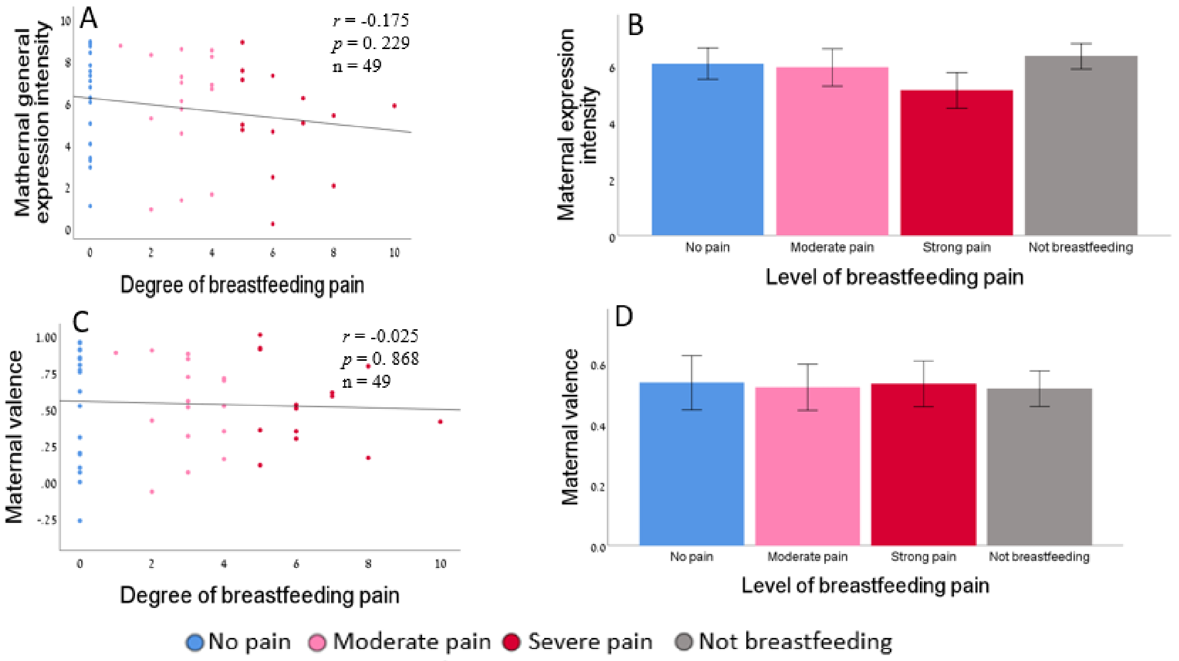

Figure S1. The association between maternal breastfeeding pain and maternal expression intensity and valence. (A) Partial Pearson correlation between the degree of breastfeeding pain and maternal expression intensity while controlling for infants' age. (B) Maternal expression intensity in the four pain groups, one-way ANCOVA, F (3,65) = 0.291 p = 0.832. (C) Partial Pearson's correlation between the degree of breastfeeding pain and maternal valence while controlling for infants' age. (D) Maternal valence in the four pain groups, one-way ANCOVA, F (3,65) = 0.844 p = 0.475.
